# Management of Femoral Shaft Fracture in Klippel-Trenaunay Syndrome with External Fixator

**DOI:** 10.1155/2016/8505038

**Published:** 2016-01-17

**Authors:** Yogendra Gupta, Ranjib Kumar Jha, Navin Kumar Karn, Sanjaya Kumar Sah, Bibhuti Nath Mishra, Manoj Kumar Bhattarai

**Affiliations:** ^1^Department of Orthopedics and Trauma, Nobel Medical College Teaching Hospital (NMCTH), Kanchanbari, Biratnagar – 5, Morang, Nepal; ^2^Department of Radiodiagnosis and Imaging, Nobel Medical College Teaching Hospital and Research Centre, Biratnagar, Nepal

## Abstract

Klippel-Trenaunay syndrome (KTS) is a rare complex malformation characterized by the clinical triad of capillary malformations, soft tissue and bone hypertrophy, and venous/lymphatic malformation. Fractures of long bones in such cases are challenging to treat. A 12-year-old female with this syndrome presented with femoral shaft fracture of right thigh. She was initially kept on skeletal traction for two weeks and then she underwent closed reduction and immobilization with external fixator with uneventful intraoperative and postoperative period. Fracture united at four and half months.

## 1. Introduction

Klippel-Trenaunay syndrome (KTS) is a rare, sporadic, complex malformation characterized by the clinical triad of capillary malformations (port wine stain), soft tissue and bone hypertrophy or occasionally hypotrophy of usually one lower limb, and venous/lymphatic malformation [1]. KTS is a mixed vascular malformation, with predominant capillary, venous, and lymphatic components, without significant arteriovenous shunting [2]. When arteriovenous malformations coexist Klippel-Trenaunay-Weber syndrome (or Parkes-Weber) can be diagnosed [3].

Fracture of long bones in such case can have unique set of problems to manage. Rarity of case itself, no definitive guidelines on management, intraoperative risk of profuse bleeding, and osteopenic bones are some factors to be taken into consideration.

We present the case of KTS with femoral shaft fracture, managed with external fixator.

## 2. Case Presentation

**Figure 1:**
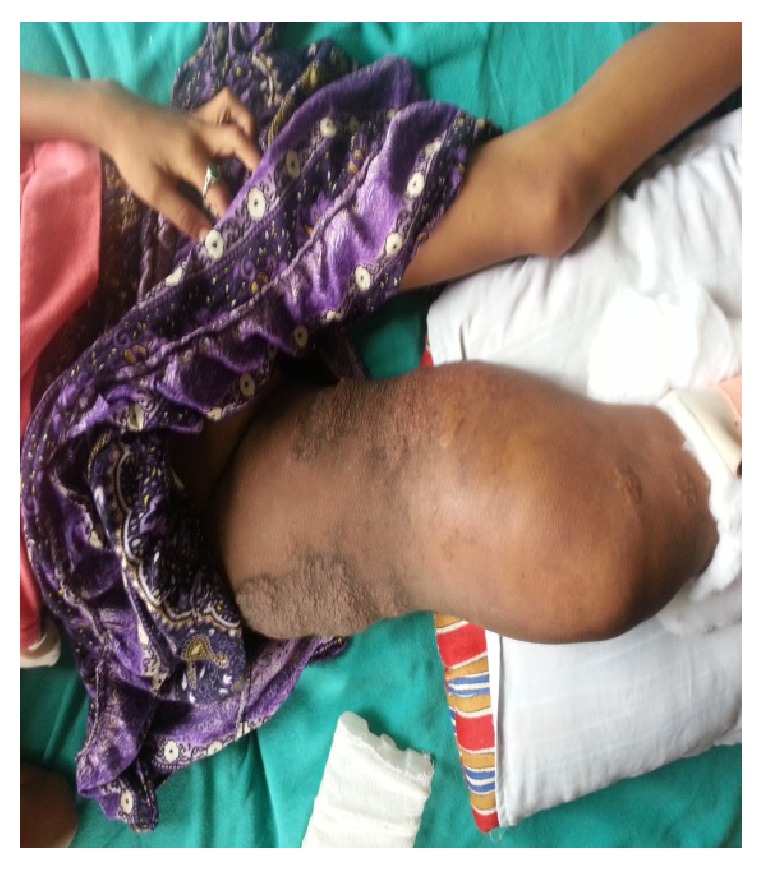
Clinical picture at the time of presentation to emergency department.

**Figure 2:**
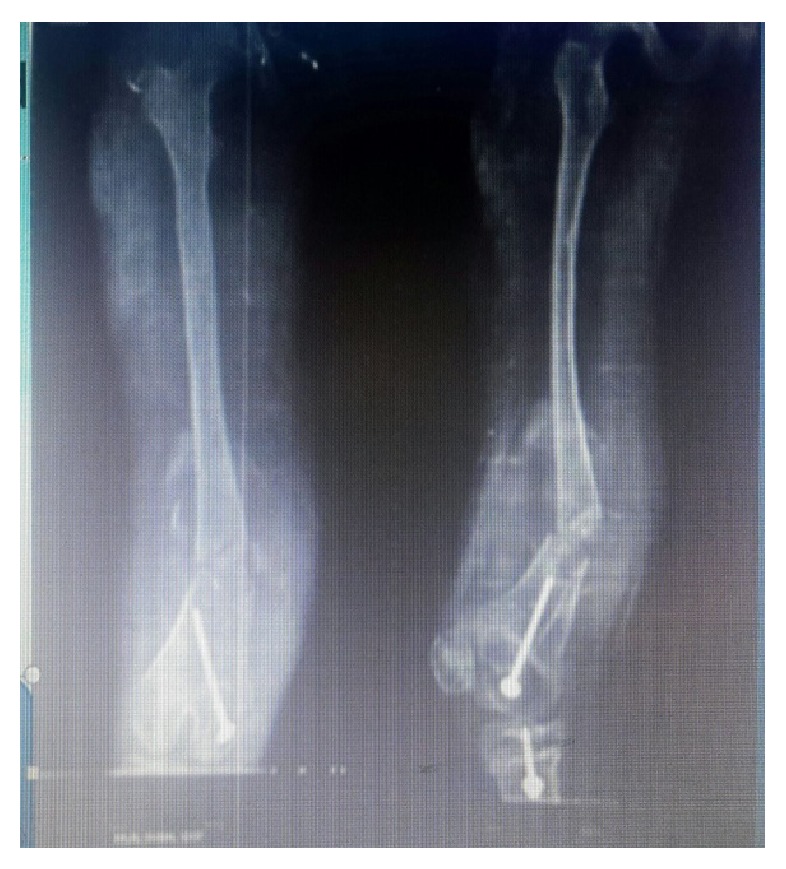
Plain X-ray of right thigh anteroposterior and lateral views showing fracture of femur at junction of middle and distal third with screws in situ used for hemiepiphysiodesis.

A 12-year-old girl came to our emergency department with pain, swelling, deformity of right thigh, and being unable to bear weight on same limb following slip and fall. She had massive swelling and multiple elevated verrucose-like lesions over thigh, the larger ones along medial and lateral aspect of thigh (Figure 1). Her distal pulses and neurology were intact. Systemic examination revealed no abnormality. She had undergone hemiepiphysiodesis of right distal femoral physis and upper tibial physis at age of seven years. X-ray of right thigh was taken and it showed fracture shaft of femur at distal third with cannulated screws in situ, which were used for epiphysiodesis (Figure 2).

Ultrasonography of the thigh was done and it showed venous malformation along whole thigh in dermis as well as in muscular planes. Contralateral thigh X-ray was taken and obvious differences in bone diameter as well as bone quality were noted.

The patient was kept on upper tibial skeletal traction for two weeks. Swelling gradually subsided. X-ray was taken with traction applied over the limb and it revealed about 15 degrees of valgus and posterior angulation with some callus at fracture site. At this time patient was taken for definitive fixation. We did closed reduction under fluoroscopy and then immobilization with external fixator. While putting the Schanz screws, distal screws were kept without predrilling due to severe osteopenia of bone.

Gradual range of motion exercise and quadriceps strengthening exercises were started from third postoperative day. Crutch walking was a bit difficult for her as her right limb was about 5 cm longer than the left.

**Figure 3:**
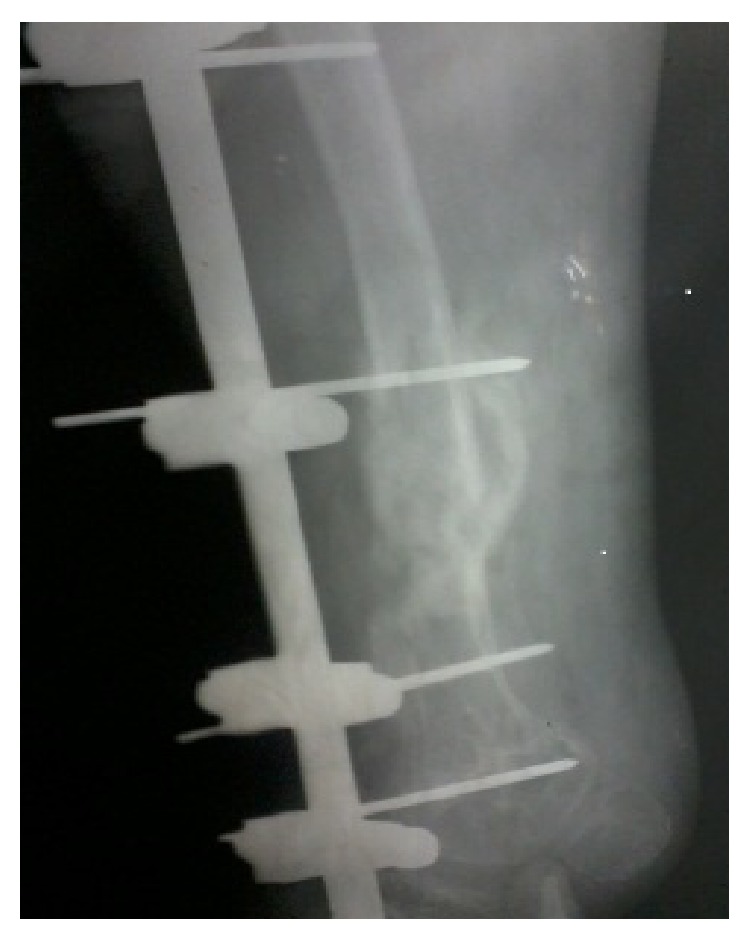
Two and half months postoperative X-ray showing good amount of callus.

**Figure 4:**
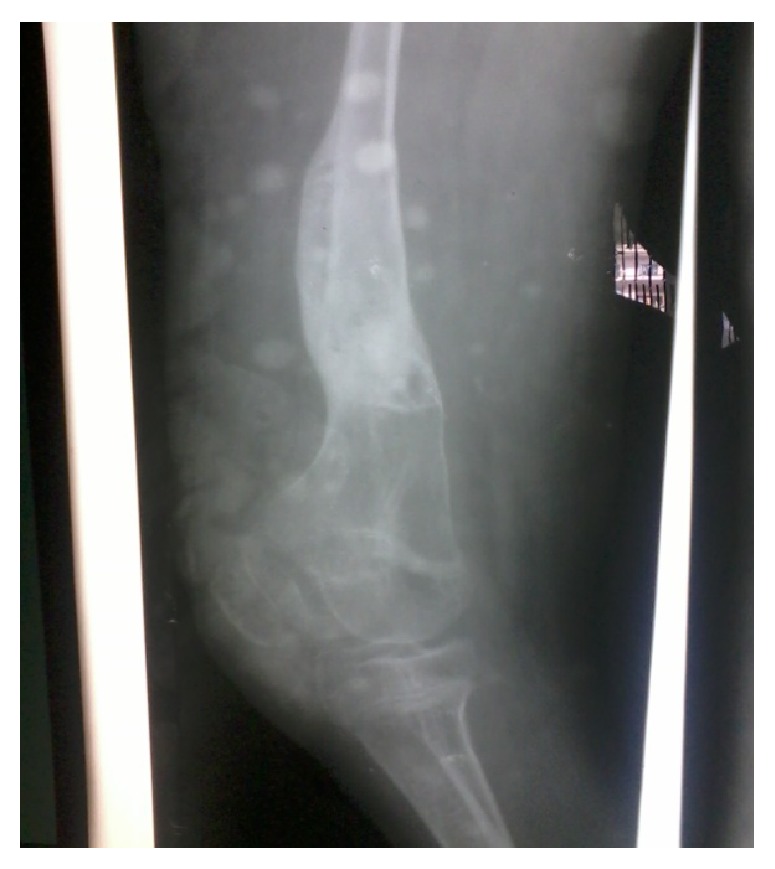
X-ray four and half months postoperative lateral view, showing fracture consolidated.

**Figure 5:**
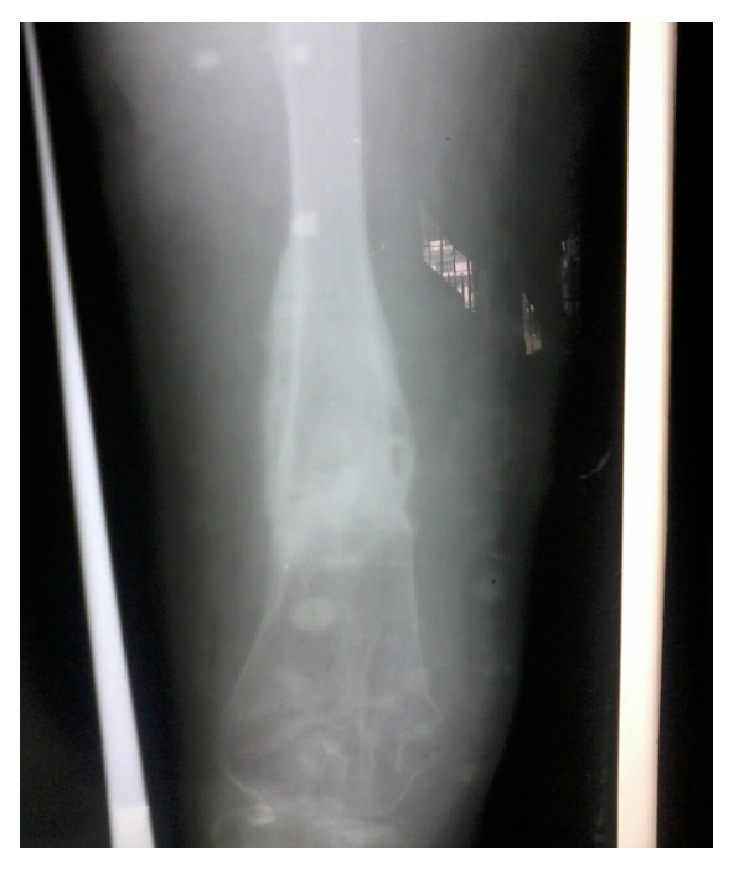
X-ray four and half months postoperative anteroposterior view, showing fracture consolidated.

**Figure 6:**
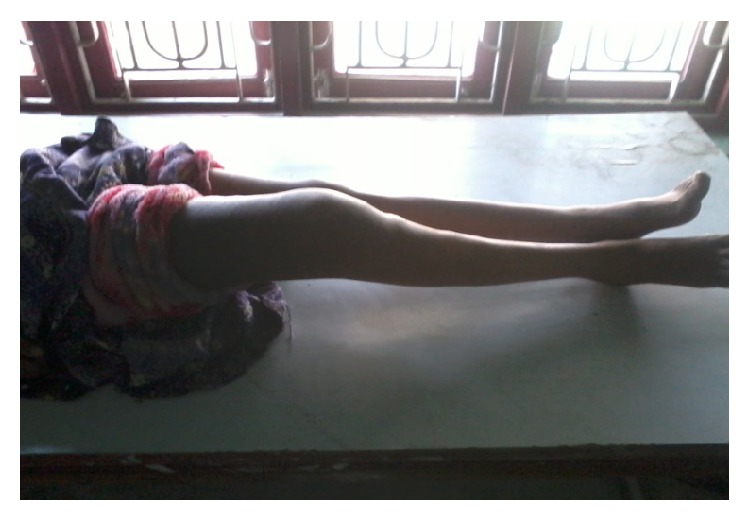
Clinical picture at final follow-up.

Follow-up X-rays showed good amount of callus (Figure 3). External fixator was removed at two and half months and partial weight bearing was started two weeks thereafter. The fracture finally united at four and half months (Figures 4 and 5). At final follow-up, at about eight months, she was doing all her daily activities independently with knee range of motion of about seventy degrees (15-degree extensor deficit and flexion up to 85 degrees) and limb length discrepancy of 5 cm (Figure 6).

## 3. Discussion

Klippel-Trenaunay syndrome is a very rare condition. It usually affects one limb but visceral involvement has also been described. The limb involvement in case of KTS is mainly due to venous and/or lymphatic malformation. This causes the soft tissue as well as the bone to become hypertrophied and the limb overall is longer than the uninvolved side. The skin and muscles contain multiple areas of venous malformation. Venous malformation in the medullary canal is also described. The bones are usually osteopenic. And these patients have high risk of fall and subsequent fracture of long bones [4].

Management of case of KTS with fracture of involved long bone is very challenging. High risk of bleeding and poor quality of bones are two important points to be taken into consideration. No definitive guidelines exit in the literature for the management of fractures in such cases. There are only few case reports in the English literature citing management of fracture in a case of KTS.

Tsaridis et al. [5] have reported the management of fracture shaft of femur in a 42-year-old female with intramedullary nail. They had to transfuse 7 units of blood intraoperatively and 3 more units in postoperative period. They also reported difficulty with locking screw due to profuse bleeding. Nahas et al. [6] also reported fracture shaft of femur in a 21-year-old male. They managed initially nonoperatively with plaster cast application. Later they did open reduction and internal fixation with locking plate. Fracture showed poor callus at one-month follow-up with backing out of implant. They applied LIPUS (low intensity pulsed ultrasound) for 10 weeks, eight minutes a day after which there was some clinical evidence of union. The final follow-up has not been reported as the patient was lost to follow-up. The other case as reported by Notarnicola et al. [7] illustrated another very similar fracture but the recovery was overshadowed by severe disabling neuropathic pain. This patient also showed complete union of the fracture one year after the accident.

In our case, we discussed each and every modality of treatment before finalizing on the way we managed. First we were very confident that union would not be problem as our patient was a child and some degree of malunion would not do any harm. Elastic intramedullary nailing was one option but we were worried about the poor bone quality. In such poor bone, inserting the nail may have caused cortical perforation. Open reduction and internal fixation with plates and screw did not appear as a good option due to the high risk of bleeding. The third option of continuing with traction followed by hip spica cast was more suitable to eliminate the complication but then it would be very cumbersome for 12-year-old female adolescent. Finally we worked to exploit benefit of both the traction and external fixation. We kept the patient on skeletal traction for about two weeks. Swelling decreased and good soft callus was formed. This helped us to correct the remaining malalignment and then the reduction was secured with external fixator. We believe that initial two weeks of traction helped us to achieve gradual reduction of the fracture. This helped us to minimize the need of repeated attempt of reduction intraoperatively and thus decreased further tissue damage. Use of external fixator helped the patient to be free from the cumbersome hip spica cast. We had kept reserve of two units of whole blood. Contradictory to previous reported cases, we did not have bleeding complication. Skin incisions were given in area free from lesions and all further dissections were made bluntly. This may have helped us in avoiding bleeding complication. Union progressed as expected. We faced challenges in rehabilitating the patient. She had preexisting limb length discrepancy and this caused difficulty in crutch mobilization. Knee mobilization was painful and the patient did not cooperate. At two-and-half-month follow-up, patient was still not using crutch properly. But fracture was healing with good amount of callus. We removed the fixator at this stage and kept patient on non-weight bearing for next two weeks. We wanted the patient to have good control over crutch walking before she put weight over the injured limb. We believe there could be early healing and better knee range of motion if patient had rehabilitated well.

To conclude, KTS is rare entity and fracture in such rare case imposes challenges to manage. Deviation from the established methods of treatment can help to minimize complications but still can give satisfactory result.
